# Efficacy and Safety of Bojungikgi-Tang for Persistent Allergic Rhinitis: A Study Protocol for a Randomized, Double-Blind, Placebo-Controlled, Phase II Trial

**DOI:** 10.1155/2022/4414192

**Published:** 2022-06-20

**Authors:** Su Won Lee, Jin Kwan Choi, Yee Ran Lyu, Won Kyung Yang, Seung Hyung Kim, Je Hyun Kim, Si Yeon Kim, Weechang Kang, In Chul Jung, Beom Joon Lee, Jun Yong Choi, Taesoo Kim, Yang Chun Park

**Affiliations:** ^1^Division of Respiratory Medicine, Department of Internal Medicine, College of Korean Medicine, Daejeon University, Daejeon, Republic of Korea; ^2^Korea Institute of Oriental Medicine, Daejeon, Republic of Korea; ^3^Institute of Traditional Medicine and Bioscience, Daejeon University, Daejeon, Republic of Korea; ^4^Clinical Trial Center, Daejeon Korean Medicine Hospital of Daejeon University, Daejeon, Republic of Korea; ^5^Department of Statistics, Graduate School, Daejeon University, Daejeon, Republic of Korea; ^6^Department of Neuropsychology, College of Korean Medicine, Daejeon University, Daejeon, Republic of Korea; ^7^Division of Allergy, Immune & Respiratory System, Department of Internal Medicine, College of Oriental Medicine, Kyung-Hee University, Seoul, Republic of Korea; ^8^Department of Internal Medicine, School of Korean Medicine, Pusan National University, Busan, Republic of Korea

## Abstract

**Background:**

Allergic rhinitis (AR) is a common disease, and conventional medications are often insufficient for treatment. Bojungikgi-tang (BJIGT) is an herbal medicine widely used in traditional medicine and has anti-inflammatory and immunoregulatory effects. We hypothesize that BJIGT would improve nasal symptoms in patients with persistent AR (PAR).

**Methods:**

This is a randomized, double-blind, placebo-controlled, phase II trial. A total of 105 patients, identified with perennial allergens, with a history of PAR and a mean total nasal symptom score (TNSS) ≥ 5 during the run-in period will be recruited from Daejeon Korean Medicine Hospital. Participants will be randomly assigned to a high-dose BJIGT group, standard-dose BJIGT group, or control group (placebo) in a 1 : 1 : 1 allocation ratio after a week run-in period. The treatment medication will be taken three times per day for 4 weeks. The primary outcome measure is the mean change in the TNSS before and after medication. The secondary outcome measures include the Korean Allergic Rhinitis-Specific Quality of Life Questionnaire, total IgE and eosinophil count, overall assessment of AR, pattern identification questionnaire for AR, and Sasang constitution. *Discussion*. The aim of this study is to investigate the efficacy and safety of BJIGT in the treatment of PAR and to determine the suitable dosage of BJIGT. Therefore, we planned a randomized, controlled, phase II trial of two different doses of BJIGT compared with placebo, and the results of this study are expected to provide evidence for the use of BJIGT as a treatment of PAR. Trial Registration. The National Clinical Trial Registry Clinical Research Information Service, CRIS, KCT0006616, https://cris.nih.go.kr/cris/search/detailSearch.do/20706.

## 1. Introduction

Allergic rhinitis (AR) is an inflammatory disease of the nasal membranes by an immunoglobulin E (IgE)-mediated response that is characterized by rhinorrhea, nasal congestion, sneezing, and nasal itching as major symptoms [1]. AR can be categorized as intermittent AR (IAR) and persistent AR (PAR) based on symptom duration. AR is a common disease with a prevalence of 10% to 40% of the global population, and it has been increasing over the last decade [2]. Moreover, it is often accompanied by eye itching, tears, postnasal drip, and fatigue, and repeated AR can be a risk factor for comorbidities such as asthma, rhinosinusitis, and nasal polyps [3]. Although AR is not a threat to life, it has a considerable impact on daily and social activities [4], eventually generating substantial medical expenses and economic burden on the public health system.

The treatment of AR typically includes allergen avoidance, pharmacotherapy, immunotherapy, and patient education [5]. It is generally centered on medication for regulating allergic inflammation and relieving symptoms because it is difficult to avoid the causes of allergens, such as pollen, spores, dust mites, and animals in daily life. Oral and topical antihistamines, intranasal corticosteroids, decongestants, intranasal cromolyn, intranasal anticholinergics, and leukotriene receptor antagonists, either alone or in combination, are used for the symptomatic treatment of AR, depending on the individual patient's situation [6]. Oral antihistamines are more effective for neutrally mediated symptoms such as itching, sneezing, and rhinorrhea, whereas intranasal corticosteroids reduce nasal congestion more noticeably in moderate-to-severe rhinitis [7, 8]. For AR that cannot be controlled using the abovementioned medications, oral corticosteroids can be used for a short period, but not as high-dose steroid injections. Topical decongestants are only recommended for short periods of within a week to prevent adverse effects, such as atrophic rhinitis [9]. Depending on the recent Allergic Rhinitis and its Impact on Asthma Guidelines, intranasal corticosteroids and intranasal H1-antihistamine are recommended for PAR patients. However, all recommendations are conditional, and treatment may depend on preference for symptom reduction vs. the avoidance of adverse effects [2]. The potential adverse effects of each treatment depend on the class of drug. For example, intranasal corticosteroids may cause headache, throat irritation, and burning; high-dose antihistamines may cause sedation; and intranasal cromolyn may cause epistaxis and sneezing [10]. Although these side effects do not affect all patients, they may become more evident with long-term use, and some patients stop taking the medication because of it. Moreover, when the medication is stopped, the effects do not last [11].

Complementary medicine has been broadly used for the treatment of AR [12], and especially in Asian countries, many trials have evaluated the efficacy of several herbal medicines [13, 14], acupuncture [15], and so on. Bojungikgi-tang (BJIGT), also known as Bu-Zhong-Yi-Qi-Tang or Hochu-ekki-to, is an herbal medication that has been widely used in traditional medicine from old times. JIGT means that “tonify the middle and augment qi,” implying strengthening the digestive system and the immune system against many kinds of infections [16] and treating debilitating conditions resulting from chronic diseases [17]. Because of these effects, BJIGT is clinically used for various diseases such as atopic dermatitis, cancer-related fatigue, and anorexia. We paid attention to the anti-inflammatory and immunoregulatory effects of BJIGT for allergic diseases. According to previous studies, in preclinical trials, BJIGT showed inhibitory effects on the increase in the number of eosinophils and protective effects of nasal mucosal tissue in the AR [18] and immune-modulatory effects including downregulating Th2 responses, decreasing IgE, eotaxin, and eosinophils in the allergic asthma model [19]. These effects may be related to the reduction in allergic symptoms. In clinical trials, the nasal symptomatic scores were significantly improved in the BJIGT treatment group and BJIGT suppressed IgE, PGE2, LTC4, and COX-2 mRNA expression in IL-4-stimulated PMN in patients with PAR [20, 21]. Several survey studies have demonstrated that BJIGT was the second most clinically used national health insurance herbal medicine for AR or PAR in Korea [22, 23]. In addition, the safety of BJIGT has been proved in the trial of 13-week oral repeated-dose toxicity [24]. However, no randomized controlled trial has evaluated BJIGT compared with placebo in the treatment of AR in Korea.

We hypothesize that BJIGT would improve nasal symptoms in patients with PAR. The aim of this study is to investigate the efficacy and safety of BJIGT in the treatment of PAR and to determine the suitable dosage of BJIGT. Therefore, we planned a randomized, double-blind, placebo-controlled, phase II trial of two different doses of BJIGT in comparison with placebo.

## 2. Methods/Design

### 2.1. Study Design

This study is a randomized, double-blind, placebo-controlled, phase II trial to evaluate the efficacy and safety of BJIGT for PAR. The trial will be conducted at the Daejeon University Daejeon Korean Medicine Hospital in Korea. We followed the methods of Lee et al. [25].

**Figure 1:**
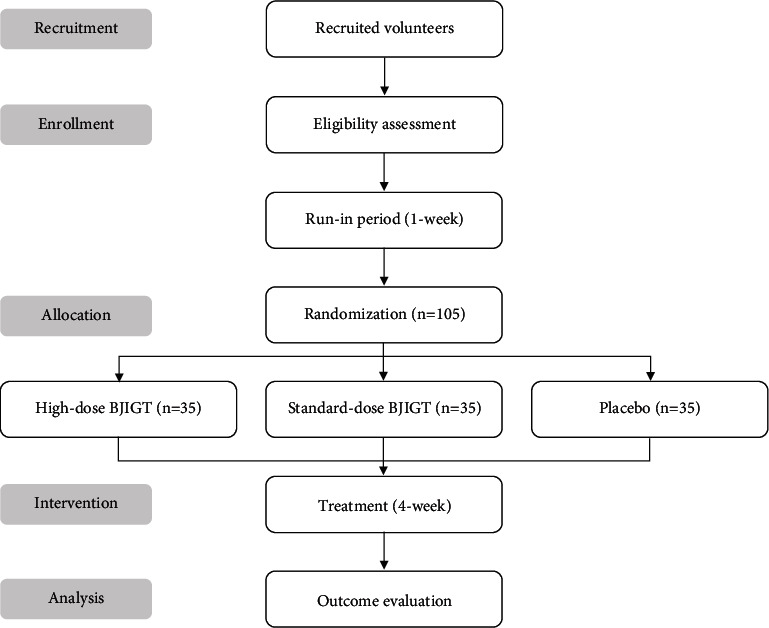
Flowchart of the study plan. BJIGT, Bojungikgi-tang.

A total of 105 participants will be recruited for this trial and randomly assigned to a high-dose BJIGT group, standard-dose BJIGT group, or control group (placebo) in a 1 : 1 : 1 allocation ratio. The trial will consist of a 1-week run-in period after screening and a 4-week treatment period. Participants will take medications three times a day for 4 weeks, with visits at 2-week intervals; the detailed study design is summarized in Figure 1 and Table 1.

### 2.2. Participants

#### 2.2.1. Inclusion/Exclusion Criteria

The inclusion criteria are as follows: (1) age 19–65 years; (2) PAR (symptoms > 4 days/week and >4 consecutive weeks) with a history of at least 2 years prior to study participation; (3) positive reactions to perennial allergens in allergy skin tests (e.g., skin prick test or intradermal test), multiple allergen simultaneous test (MAST), or Immuno CAP that were conducted within 12 months. The criteria for positive responses to specific allergens are defined as follows—prick tests: a wheal size of ≥3 mm compared with the diluent control; intradermal tests: a wheal size of ≥7 mm compared with the diluent control; MAST or Immuno CAP: ≥ class 2; (4) average daily reflective total nasal symptom score (r-TNSS) of ≥5 at the third visit during the run-in period; (5) the ability and willingness to record subject diaries; (6) equally maintain the surrounding environment during the entire period of the clinical study; and (7) voluntarily agreed to participate in this clinical study in written form.

The exclusion criteria are as follows: (1) nonallergic (angioneurotic, infectious, and drug-induced) rhinitis; (2) presence of asthma; (3) clinically significant nasal deformities, such as obstructive nasal polyps or nasal septum; (4) experienced damages around the nasal cavity or surgeries within 12 weeks; (5) a past medical history of acute or chronic sinusitis within 4 weeks; (6) initiated immunotherapy or changes in dosage within 4 weeks; (7) experienced use of long-acting antihistamines; (8) upper respiratory tract infections, including the common cold, and systemic infections within 2 weeks; (9) comorbidities or clinical significant disorders of the kidney, liver, psychiatric system, cardiovascular system, respiratory system, endocrine system, and central nervous system, which may interrupt the treatment of cancers, the safety assessment, or completion of this clinical study; (10) abnormal renal function (creatinine values ≥ 2 times the upper limit of normal); (11) severe abnormal liver function tests (alanine transaminase (ALT) or aspartate transaminase (AST) values ≥ 2 times the upper limit of normal); (12) hepatitis A (active), hepatitis B (active), or hepatitis C; (13) unregulated hypertension (systolic blood pressure > 180 mmHg or diastolic blood pressure > 100 mmHg); (14) electrolyte abnormalities (potassium test readings outside the normal range); (15) hypokalemia, administering drugs (such as furosemide, thiazide, amphotericin, cisplatin, and digitalis), or a condition that can show hypokalemia (such as hypomagnesemia, Bartter syndrome, and Gitelman syndrome); (16) anticoagulants (heparin, warfarin, aspirin, etc.) for cardiovascular disorders or blood coagulation disorders; (17) blood coagulation abnormalities (PT-INR and aPTT readings outside the normal range); (18) the use of concomitant medications or those who are expected to require the use of prohibited concomitant medications during the period of the clinical study a minimal period following the use of concomitant medications has passed (3 days for nasal passages, topical, eye drops, or systemic antihistamines (10 days for long-acting agents, such as loratadine, fexofenadine, and cetirizine; 14 days for ketotifen; and 3 months for astemizole); 14 days for cromolyn nasal or nedocromil; 3 days for anticholinergics; 3 days for nasal or systemic decongestants; 30 days for nasal or systemic corticosteroids; 3 days for reserpine; 3 days for antitussives and expectorants; 7 days for leukotriene modifiers; 14 days for systemic antibiotics; 2 days for nonsteroidal anti-inflammatory drugs (NSAIDs) (a low-dose aspirin below 100 mg can be used); and 14 days for traditional herbal medicinal products that may affect rhinitis and eye symptoms); (19) chronically used tricycle antidepressants, beta-agonists, and bronchodilators that may affect the efficacy of test drugs; (20) r-TNSS during the baseline period within 1 week of the third visit was recorded for less than 4 days; (21) plan of long-term movement to other areas during the period of the clinical study; (22) history of excessive alcohol use or drug addiction; (23) history of hypersensitivity reaction to active ingredients or excipients of the investigational product; (24) genetic disorders, such as galactose intolerance, Lapp lactase deficiency, or glucose-galactose malabsorption; (25) subjects who did not agree to use contraception by medically permitted methods (e.g., surgical treatment of infertility, intrauterine device, condom or diaphragm, and injectable or insertable contraceptives) during the period from dosing of the investigational drug to 90 days after the clinical study among female subjects of childbearing potential and male subjects with a female partner; (26) pregnant or lactating women; (27) participation in a different clinical study within 4 weeks before participation in this trial; and (28) subjects who were determined to be ineligible to participate in this trial.

To determine whether the rhinitis is allergic, potential participants will be examined using MAST at the screening, and participants classified as class 2 or higher in the MAST that tests 108 types of allergens will be enrolled. Each patient's enrollment will be determined by the investigator, a Korean medicine doctor who treats allergies and respiratory diseases. During the trial period, the participant can voluntarily withdraw at any time or can be dropped at the discretion of the researcher.

#### 2.2.2. Sample Size

The number of subjects is evaluated on the basis of detecting significant differences in the primary outcome, mean change of TNSS before and after medication among the groups with AR patients. In an earlier clinical study that evaluated other herbal medicines compared with placebo, the difference in TNSS was reported to be 0.97 with a standard deviation of 1.3 [14]. With a power of 80% (1–*β*) and a significance level of 5% (*α*), the target sample size is 29 patients per group. Considering an assumed dropout rate of 20%, a total of 105 subjects (35 subjects per group) will be required. Similarly, the number of subjects in previous exploratory phase II trials ranged from 20 to 42 per group [26–28].

#### 2.2.3. Recruitment

Participants are being recruited through advertising, and posters will be displayed in the outpatient departments of the hospital and the website homepage of the hospital. The posters will contain simple explanations of the enrollment criteria, purpose of the study, intervention, and predictable adverse events. Written informed consent will be obtained ahead of enrollment at our hospital's clinical trial center, and participants can determine whether or not to participate in the trial.

### 2.3. Interventions

The participants will receive placebo for a 1-week run-in period and high-dose BJIGT or standard-dose BJIGT or placebo for the 4-week treatment period. BJIGT and placebo granules are sealed in aluminum bags, and the participants will be instructed to take two packets of medicine (5.0 g) with water three times per day after each meal. The dosage is determined to be 15 g per day for the high-dose BJIGT and 7.5 g per day for the standard-dose BJIGT according to the Korean Ministry of Food and Drug Safety (MFDS) approval and the pharmacologically active dose in expectorant effective tests [29].

The BJIGT to be used in this study is composed of dried brown granules, and the extract is allowed and controlled by the Korean MFDS. Hankookshinyak Corporation (Nonsan, Korea) manufactures the BJIGT and placebo in accordance with Korea Good Manufacturing Practice standards. A 2.5 g sample of BJIGT consists of ten medicinal herbs: Ginseng Radix 0.665 g, Atractylodis Rhizoma 0.665 g, Astragali Radix 0.665 g, Angelicae Gigantis Radix (0.5 g), Citri Unshius Pericarpium 0.335 g, Bupleuri Radix 0.335 g, Zizyphi Fructus 0.335 g, Glycyrrhizae Radix 0.25 g, Cimicifugae Rhizoma 0.165 g, and Zingiberis Rhizoma 0.085 g. The placebo does not include any of these active ingredients and is made of lactose, corn starch, caramel coloring, etc. Both granules had the same shape, weight, and color as gray brown.

The participants will record their symptoms twice daily—morning and evening—in their TNSS diary for a total of 5 weeks. To improve and evaluate compliance, participants will be requested to record their dosage and return the remaining medications at the third, fourth, and fifth visits. Additional therapeutic interventions for AR equivalent to the exclusion criteria of the protocol will not be permitted during the trial.

### 2.4. Outcome Measures

#### 2.4.1. Primary Outcome Measure

*TNSS*. The primary outcome in this study is the difference in the changes in TNSS before and after the medication among the three groups. TNSS is the most widely used outcome in patients with AR and evaluates symptoms of rhinorrhea, nasal congestion, nasal itching, and sneezing. Each symptom will be rated on a four-point severity scale (0 = no symptoms, 1 = mild, 2 = moderate, and 3 = severe), and the total score will range from 0 to 12. There are two types of TNSS: reflective TNSS (r-TNSS), which evaluates how they felt during the past 12 hours, and instantaneous TNSS (i-TNSS), which evaluates how they feel at the time of assessment [30]. Participants will record the r-TNSS and i-TNSS in the diary twice daily during the run-in period and study period. The baseline data will be the average of the symptom scores during the run-in period, and mean score changes for 4 weeks or 2 weeks compared with baseline will be analyzed.

#### 2.4.2. Secondary Outcome Measures

*KARQLQ*. Measurement of AR-specific quality of life (QOL) impairment is significant for the assessment of the burden and efficacy of treatment. We will use the Korean Allergic Rhinitis-Specific Quality of Life Questionnaire (KARQLQ), which is the validated QOL questionnaire for Korean AR patients that consists of 15 questions about activities, nasal symptoms, practical problems, and other symptoms with a 5-point scale [31]. The KARQLQ will be measured at the third, fourth, and fifth visits (weeks 0, 2, and 4).

*Total IgE and Eosinophil Count*. Total IgE and eosinophil counts will be examined at the third and fifth visits (weeks 0 and 4) to assess the impacts of BJIGT on allergic and inflammatory responses. BJIGT has been demonstrated to have antiallergic and anti-inflammatory effects in animal studies and several clinical studies [18, 21].

*Overall Assessment of Allergic Rhinitis*. The overall degree of symptom changes for AR will be evaluated after treatment (fifth visit, week 4). The following scoring method will be used: 0 = very much improved, 1 = much improved, 2 = slightly improved, 3 = no change, 4 = slightly worse, 5 = much worse, and 6 = very much worse [32].

*Pattern Identification Questionnaire for AR*. To seek a more precise use of BJIGT, we will use the pattern identification (PI) questionnaire for AR based on a traditional Korean medicine perspective. The PI questionnaire for AR V.3.0 was developed by specialists in the Department of Otolaryngology of Korean medicine [33]. This questionnaire sorts patients with AR as possessing lung-heat, lung-cold, or spleen qi deficiency using the nasal symptoms and general conditions of AR patients.

*Sasang Constitution*. The Sasang constitution system by constitutional typology in Korean medicine differentiates individuals into four constitutional types. We will use the revised questionnaire for the Sasang Constitution Classification (QSCC II+) to refer to the differences in the effects of the Sasang constitution [34].

#### 2.4.3. Safety Assessment

Safety will be assessed through laboratory examinations, vital signs, and adverse events (AEs). To observe the safety of the participants, complete blood cell counts, blood coagulation test, blood chemistry examination (liver and kidney function test), serum hepatitis test, and urine test will be conducted at screening and at the fourth and fifth visits. The investigator will check vital signs and any unexpected or expected AEs at every visit and record details on a case report form (CRF). If serious AEs occur, the investigator will promptly stop the subject from taking medication and report it in accordance with the guidelines of the IRB.

### 2.5. Assignment of Interventions

#### 2.5.1. Allocation

The randomized allocation sequence will be produced by an independent statistician using SAS version 9 statistical software (SAS Institute, Cary, NC). The subject who meets the criteria will be given an identification code (random assignment number) with the same allocation ratio of 1 : 1 : 1 for a high-dose BJIGT, standard-dose BJIGT, or placebo group. The statistician will keep the randomization table separately until the blinding of the trial is maintained. The assignment is planned according to the assignment table created by a random assignment method that can be specifically designed and reproduced in advance. The statistician manages the assignment table, and the file is kept closed-door.

#### 2.5.2. Blinding

The subjects will be assigned to a random number and receive the medication package corresponding to the participant's identification code. Because this trial is double-blind, the participants or investigators will not be conscious of group assignment until the trial is ended. Removal of blindness will only be allowed in cases of serious medical emergencies.

### 2.6. Data Management and Monitoring

The investigators will obey the standard procedures of the trial. The investigators who assess outcome measures should be regulated to physicians who have attended training meetings. Data collection will be conducted at every visit for all groups. Collected documents and data on CRFs will be handled by only the principal investigator or those who are permitted to access the data. The investigator should preserve a copy of all clinical trial-related reports, the subjects' records, consent, and case records for at least three years after the end of the trial in a controlled-access laboratory archive.

Clinical trial monitoring will be performed via regular visits and occasional telephone calls by a clinical research associate (CRA) once or twice monthly. The CRA will confirm whether the trial is being executed in compliance with the protocol, and AEs are being appropriately reported and recorded on CRFs. The monitor will inspect the entire process of the clinical trial, and matters will be discussed with the investigator as and when they appear.

### 2.7. Statistical Analysis

An independent statistician will analyze the data using SAS Analytics Pro. A full analysis set (FAS) is defined as the analysis based on intention-to-treat (ITT) principles, including the subjects who received at least one trial intervention when evaluating effectiveness. In this trial, efficacy evaluation will be primarily based on ITT analysis, and per-protocol (PP) analysis will be applied as a secondary analysis, including the subjects completing the entire trial without violating the protocol.

The demographic characteristics and baseline data will be expressed as means and standard deviations (SD) for continuous variables, and frequencies and percentages for categorical variables. The baseline differences between groups will be compared using an independent *t*-test for continuous values and the *χ*^2^ test or Fisher's exact test for categorical values. The efficacy outcome measures and the mean differences of TNSS from baseline to the treatment period will be analyzed by analysis of covariance (ANCOVA) with baseline data as a covariate for linear mixed models (LMMs). The baseline data are defined as the mean TNSS per day in the run-in period and will be compared with the diary-based mean TNSS for the 2-week treatment period or 4-week treatment period. The efficacy analysis methods are the same for TNSS by PI and Sasang constitution, KARQLQ, and biomarkers. The overall assessment of AR after the trial will be analyzed using a general linear model. All analyses will be set at a 5% significance level.

Safety evaluation will be conducted in the subjects who took one or more interventions, and the investigator will confirm at least one safety-related data set by visit or call after the intervention. A comparison of the number of AEs related with the trial will be conducted using the Kruskal–Wallis test, and group comparisons of the proportion of subjects who experienced one or more AEs will be analyzed using Pearson's *χ*^2^ or Fisher's exact test.

## 3. Ethics and Dissemination

This clinical trial protocol follows all relevant regulations, including the Helsinki Declaration, ICH GCP Guidelines, and Consolidated Standards of Reporting Trials guidelines. This study was permitted by the MFDS on investigational new drug (IND) application in May 2021 and by the Institutional Review Board (IRB) of the Daejeon University Daejeon Korean Medicine Hospital (approval number: DJDSKH-20-DR-13) in June 2021, including the protocol, written patient consent, consent form, and patient registration process (e.g., advertising). This study is registered at the National Clinical Trial Registry Clinical Research Information Service (https://cris.nih.go.kr) with the identifier number KCT0006616 in July 2021.

Before the trial, the investigators will provide all the information relevant to the advantages and disadvantages of participating in this study through a written consent form, and the subjects will sign a document comprising all the instructions for the subjects. Participants will be paid a small transportation fee for their participation in this trial. After the subjects decide whether to participate and sign the document, all patient identifiable data will be kept confidential in a controlled-access laboratory archive. The clinical trial documents will be recorded and differentiated by the subject identification code throughout the trial, and monitors and inspectors associated with this clinical trial have access to the subject's records for the reason of monitoring and managing the progress of the trial.

## 4. Discussion

AR is a common disease with a high prevalence and significantly affects QOL [3]. AR is usually prolonged, but conventional medications currently used for AR have adverse effects in some patients, especially in long-term use. Antihistamines may cause sedation, fatigue, and impaired mental status and are less effective for nasal congestion [10]. Intranasal corticosteroids have adverse effects, including headache, epistaxis, throat irritation, and nasal dryness, and decongestants are effective for nasal obstruction, but usage for more than one week may induce drug tolerance [35]. Therefore, other additional medicines that can be used long term are required.

BJIGT is a widely known herbal medication in various Asian countries and is used for the treatment of allergic diseases. In previous studies, BJIGT showed anti-inflammatory and immunoregulatory effects by suppressing eosinophils, IgE antibody, cytokines, and histamine related to early-phase response and late-phase response of AR, leading to clinical improvement [18–21, 36]. These effects support that BJIGT may be beneficial for patients with AR.

Although BJIGT is an herbal drug that has already been clinically used for AR, there have been no randomized controlled trials for Korean patients with PAR. This study will support evidence for the efficacy and safety of BJIGT in a standard design and raises objective grounds for Korean medicine used in clinical practice. Further, we expect that BJIGT with a suitable dosage is recommended as a complementary medicine for PAR.

## Figures and Tables

**Table 1:** Schedule of enrollment, interventions, and outcome measurements.

Time point, wk	Screening	Run-in period	Study period		
	−2 W	−1 W	0 W	4 W	8 W
Visit	1	2	3	4	5
Enrollment					
Informed consent	X				
Eligibility screen	X	X			
Allocation			X		
Demographic characteristics	X				
Medical history	X				
Chest X-ray, EKG	X				
Vital signs	X	X	X	X	X

Interventions					
Prescription		X	X	X	
High-dose BJIGT					
Standard-dose BJIGT					
Placebo					

Assessments					
Clinical laboratory test^(1)^	X			X	X
Pregnancy diagnosis test	X				X
MAST	X				
TNSS					
KARQLQ			X	X	X
Biomarkers^(2)^			X		X
Overall assessment					X
Pattern identification			X		
Sasang constitution			X		
Compliance			X	X	X
Adverse reaction assessment		X	X	X	X

(1) complete blood cell count tests, blood coagulation test, blood chemistry examination (liver and kidney function test), serum hepatitis test, and urine analysis; (2) total IgE, eosinophil count. BJIGT, Bojungikgi-tang; MAST, multiple allergen simultaneous tests; TNSS, total nasal symptoms score; KARQLQ, Korean Allergic Rhinitis-Specific Quality of Life Questionnaire.

## Data Availability

The data sets used or analyzed during the current study will be available from the corresponding author on reasonable request.
